# An open source isolated data acquisition with trigger pulse generation for ion mobility spectrometry

**DOI:** 10.1016/j.ohx.2024.e00600

**Published:** 2024-10-28

**Authors:** Tim Kobelt, Martin Lippmann, Alexander Nitschke, Lou Kielhorn, Stefan Zimmermann

**Affiliations:** Leibniz University Hannover, Institute of Electrical Engineering and Measurement Technology, Department of Sensors and Measurement Technology, Appelstr. 9A, 30167 Hannover, Germany

**Keywords:** Ion mobility spectrometry, Data acquisition, Pulse generation, Optical isolation

## Abstract

Ion mobility spectrometers (IMS) are used in a wide variety of applications, including trace gas detection in safety and security applications, but also in more analytical applications, e.g., in medicine or food quality monitoring. Consequently, IMS are often coupled with other separation techniques and laboratory equipment, requiring synchronization between the external equipment and the IMS electronics. In addition, IMS and the associated electronics are becoming increasingly complex due to ongoing instrumental developments. In this work, we present an open source data acquisition hardware tailored to the requirements of advanced IMS, but also applicable to other applications. The data acquisition hardware provides trigger pulses for synchronized operation of the IMS ion gate or external devices. In addition, the data acquisition hardware allows for parallel digitalization using two isolated 16-bit analog-to-digital converters (ADC) with up to 250 kilosamples per second. The galvanically isolated trigger input ensures a synchronized start of the IMS measurements, particularly when connecting external instrumentation such as a gas chromatograph. Furthermore, due to the isolated ADCs, the hardware allows great flexibility in defining the ground potential of the instrument setup.

## Graphical abstract

**Figure:**
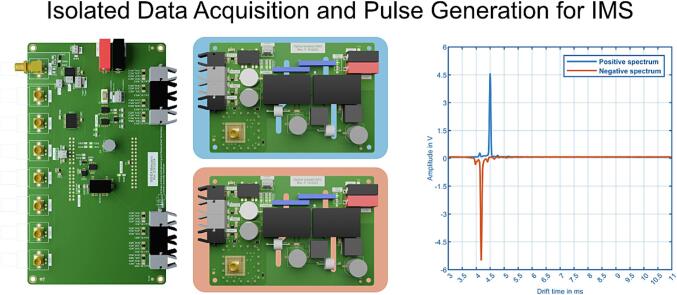

**Specifications table**

**Table:** 

Hardware name	Isolated data acquisition with trigger pulse generation
Subject area	• Engineering and materials science • Chemistry and biochemistry • Educational tools and open source alternatives to existing infrastructure
Hardware type	• Measuring physical properties and in-lab sensors • Electrical engineering and computer science
Closest commercial analog	No commercial analog is available
Open source license	CC BY-SA 4.0
Cost of hardware	1,000 €
Source file repository	https://doi.org/10.17632/5wh9bdmnm5.1

## 1 Hardware in context

Ion mobility spectrometers (IMS) are highly sensitive gas detection devices [1] that are mainly used in safety and security applications for the detection of toxic industrial compounds [2], [3], [4], explosives [5], [6] and chemical warfare agents [7], [8]. Furthermore, IMS are increasingly used in a variety of other fields, including food quality control [9], [10], [11], human breath analysis [12], [13], [14], and drug screening [15], [16], [17]. IMS offer highest sensitivity, but suffer from comparatively low spectral resolution and chemical cross-sensitivities, so that IMS are often combined with gas chromatographs (GC) to add a second dimension of separation, especially when analyzing more complex samples [18]. However, coupling with additional instruments requires specific timing and synchronization of instruments. For example, in the case of coupling with a GC, consecutive IMS spectra are recorded to resolve the GC chromatogram. For this reason, the ion gate of the IMS must be triggered together with the data acquisition and the interval between two spectra has to be constant. Furthermore, it is crucial that the data acquisition starts together with the beginning of the GC run and each recorded spectrum is transferred to the measurement software in correct order.

The hardware presented here allows for the acquisition and processing of ion mobility spectra and the generation of synchronized trigger pulse patterns for IMS operation. In IMS it is often beneficial to define the ground potential at the complex shutter electronics [19] or the ion source [20], [21]. Therefore, the detector of the IMS is at a high potential in some instruments [20], [21]. In addition, for dual polarity IMS [22], two analog-to-digital converters (ADCs) must sample the output signals in parallel at high potential to obtain both ion polarities simultaneously. However, most commercial analog ADCs are referenced to ground. To overcome these limitations, the ADCs have to be optically isolated. No commercial product or existing open source hardware addresses all these requirements. However, it is worth noting, that a commercially available solution for parallel readout of two amplifiers at high reference potential is an oscilloscope with two optically isolated probes. Unfortunately, an oscilloscope would not offer 16-bit resolution, but only 8 to 12 bits, depending on the price. In addition, a pulse generator would be required to generate the shutter pulses. However, this solution is far more expensive than the presented hardware. The presented hardware simplifies the development of IMS research instruments or lab demonstrators without being limited by the technical barriers of fast and synchronized data acquisition, data processing, and precisely timed trigger pulse generation.

## 2 Hardware description

**Fig. 1:**
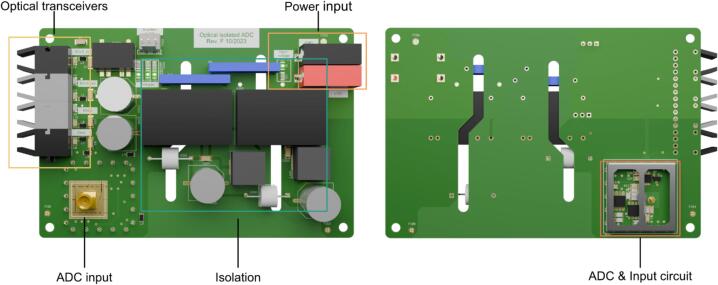
Top view of the ADC board (left) and bottom view of the ADC board (right).

This work presents an isolated data acquisition based on two simultaneous sampled 16-bit ADCs of type LTC1864 (Analog Devices) and the synchronized generation of trigger pulse patterns required for IMS operation. The hardware is based on an XC7Z020 (Xilinx) system on a chip (SoC) containing two Arm Cortex A9 and a Xilinx Artix Programmable Logic. For simplification, a commercially available evaluation board (Digilent Arty Z7-20) containing the SoC, DDR3-Random-Access memory (Ram), Ethernet Physical Layer (PHY), and power supply for the SoC is used as a platform. The schematics and board files provided with this publication contain all necessary components for the optical isolation of the two ADCs as well as electrical inputs and outputs for trigger pulse generation. The isolation of the Serial Peripheral Interface (SPI) of the ADCs has been described in [23] and follows the article from Analog Devices [24]. The ADC board and data acquisition board are connected via fiber-optic cables by optical transceiver and receiver of type AFBR-1624Z and AFBR-2624Z (Broadcom Limited). The hardware design for an isolation of up to 12 kV of the ADC is shown in Fig. 1 and is included in the provided files. In this design, two Recom REC6-1215DRW/R10/A/X1 DC/DC converters are used in series for isolated power supply. The board supports an input signal voltage range between −12 V and +12 V and provides an isolated supply voltage of + −15 V for the IMS current amplifier. For example, the open source transimpedance amplifier from Reinecke et. al [25] could be used. Therefore, the isolation voltage is only limited by the isolation of the supply voltage of the ADC and the current amplifier. For higher isolation voltages a topology such as in [26] can be used. It is also conceivable to use a rechargeable battery for the power supply of the ADC and current amplifier. The maximum isolation voltage of the data transmission can be set as high as required by selecting sufficiently long fiber-optic cables.

**Fig. 2:**
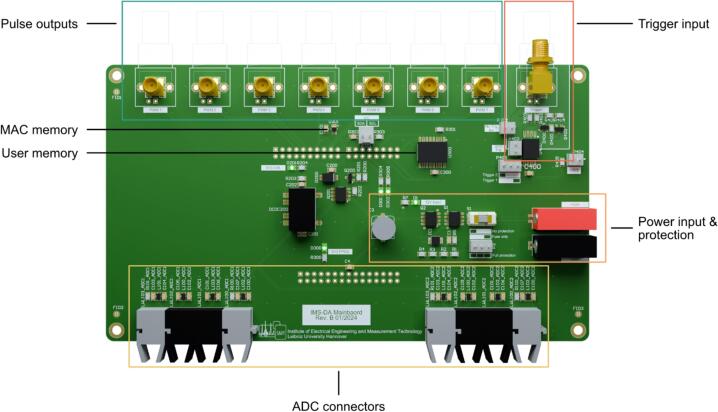
Top view of the data acquisition mainboard with highlighted functional sections.

The data acquisition mainboard shown in Fig. 2 supports two parallel ADCs of type LTC1864. The ADCs can sample with up to 250 kilosamples per second (ksps) at 16-bit resolution, and are synchronized with the trigger pulse generation. This ensures that, for example, the trigger pulse, controlling the ion gate of the IMS, is synchronized with the acquired ion mobility spectra. The interface of the ADCs is implemented in the field programmable part of the SoC and processes the two ADC data streams in parallel. The ADC-unit includes several watchdog timers that ensure correct operation even when a bit flip occurs in the high frequent SPI signal transmitted from the ADCs. This is especially beneficial for devices with fast-switching high electrical fields such as FAT-IMS [27] or ultra-fast polarity switching IMS [28]. However, no further electromagnetic interference tests have been performed. By using these watchdog timers, precise timing of each sample point is always guaranteed. In addition to the ADC interface, all time-critical tasks are also implemented in the programmable logic. The data processing, communication with the computer, and configuration of the programmable logic are handled in the ARM-cores.

The trigger pulse generation provides up to seven independent pulse channels with a time resolution of 10 ns. In addition to single pulses with pulse “high” time and pulse “low” time, pulse patterns can be generated using the additional seven burst pulse generators and the logical combination of individual pulse channels. All blocks in the Field Programmable Gate Array (FPGA) are synchronized by the configurable period clock, which controls the start of each IMS cycle. The recorded spectra are buffered inside the 512 Mbyte DDR3 RAM. Especially for GC-IMS applications or IMS with high repetition rates [29] the presented hardware ensures that each spectrum is recorded with precise timing and is stored in the implemented ring buffer for transmission to the computer. An incremental number is assigned to each spectrum by the FPGA to generate a relative timestamp for each spectrum. Each spectrum consists of up to 16,000 sample points, which results in a maximum sampling time of 64 ms per spectrum at the highest sampling frequency of 250 ksps. With the optional sampling delay (start delay of the data acquisition after the trigger pulse) and the adjustable sampling frequency, it is possible to acquire even longer IMS spectra. In addition, this allows sampling only that part of a spectrum that contains data. The acquisition hardware can also low-pass filter and average the recorded IMS spectra, which makes post-processing of the measurement data obsolete. The filtered and averaged spectra are sent to the computer over ethernet using Transmission Control Protocol / Internet Protocol (TCP/IP) [30], which ensures error-tolerant communication between the computer and the acquisition hardware. TCP/IP initially relies on a handshake between sender and receiver, after the connection has been established, received messages are acknowledged by the receiver so that the sender knows exactly whether a message has arrived. If a sent message is not acknowledged, it is automatically resent. In addition, sequence numbers can be used to correct the order of incoming packets at the receiver and checksums guarantee the correct content of the packet. The acquisition hardware is fully compatible with Dynamic Host Configuration Protocol (DHCP) [31] and can be integrated easily into existing laboratory network structures. If a direct connection between the acquisition hardware and the computer is required, the acquisition hardware can be accessed at a fixed IP address.

Synchronization with external instruments such as lasers [32], [33], gas chromatographs, or mass spectrometers can either be accomplished by using the trigger pulse output or utilizing the isolated trigger input of the acquisition hardware. This trigger input is galvanically isolated and offers a flexible range of the high level of the input signal between 4 V to 32 V. The trigger signal is routed into the FPGA and can be used for different trigger modes. For example, it is possible to trigger each IMS spectrum or to start a series of IMS spectra.

The whole system can be powered by a single 12 V power supply with minimal 30 W and is protected by an optional power protection circuit.

The hardware presented in this work provides a complete data acquisition platform for IMS and other applications. It includes:

• Two synchronized error-tolerant isolated ADCs with 250 ksps and 16-bit resolution
• Up to seven independent but synchronized trigger pulse outputs with 10 ns time resolution
• Buffered data transition over ethernet using TCP/IP
• Galvanic isolated trigger input to synchronize IMS with external hardware with trigger delays in the low 100 ns

## 3 Design files summary

**Table:** 

Design file name	File type	Open source license	Location of the file
*Mainboard.pdf*	*Schematics of the mainboard*	*CC BY-SA 4.0*	*MendeleyData*
*ADC-Board.pdf*	*Schematics of the ADC-board*	*CC BY-SA 4.0*	*MendeleyData*
*PCB-Project.zip*	*PCB Altium projects of the mainboard and ADC-board*	*CC BY-SA 4.0*	*MendeleyData*
*Gerber.zip*	*Production files of the mainboard and ADC-board*	*CC BY-SA 4.0*	*MendeleyData*
*Firmware.zip*	*FPGA and microcontroller firmware source code*	*CC BY-SA 4.0*	*MendeleyData*
*Fsbl.elf*	*First stage bootloader for the SoC*	*CC BY-SA 4.0*	*MendeleyData*
*BOOT.bin*	*Firmware binary for the SoC*	*CC BY-SA 4.0*	*MendeleyData*
*Parameters.xlsx*	*Table of operating parameters*	*CC BY-SA 4.0*	*MendeleyData*
*TCP-Protocol.docx*	*Documentation of the communication protocol*	*CC BY-SA 4.0*	*MendeleyData*

## 4 Bill of materials summary

**Table:** 

Designator	Component	Number	Cost per unit	Total cost	Source of materials
*Mainboard PCB*	*Printed Circuit Board*	1	35,64 €	35,64 €	https://www.multi-circuit-boards.eu
*C1*	*Capacitor SMD 0805 2n2 50 V NP0*	1	0,21 €	0,21 €	https://www.mouser.de/c/?q=81-GRM215C1H222JA01J
*C3*	*Electrolyte-Capacitor SMD 330uF 35 V*	1	0,65 €	0,65 €	https://www.mouser.de/c/?q=661-EMZR350ARA331MHA
*C4, C100_ADC1, C100_ADC2, C101_ADC1, C101_ADC2, C102_ADC1, C102_ADC2, C103_ADC1, C103_ADC2, C104_ADC1, C104_ADC2, C105_ADC1, C105_ADC2, C106_ADC1, C106_ADC2, C107_ADC1, C107_ADC2, C200, C201, C202, C300, C301, C400*	*Capacitor SMD 0805 10uF 35 V X5R*	23	0,23 €	5,31 €	https://www.mouser.de/c/?q=810-C2012X5R1V106K1C
*D1, D201, D300, D301, D302*	*LED SMD LED yellow*	5	0,14 €	0,72 €	https://de.farnell.com/search?st=1318247
*D200*	*Diode SMD US1M-E3/5AT*	1	0,09 €	0,09 €	https://www.digikey.de/de/products/detail/vishay-general-semiconductor-diodes-division/US1M-E3-5AT/2149381
*D400*	*Zener Diode SMD BZX84Z 5 V1*	1	0,29 €	0,29 €	https://www.mouser.de/c/?q=621-BZX84C5V1W-F
*DCDC200*	*DC/DC Converter SMD TSRN 1-2450SM*	1	10,27 €	10,27 €	https://www.digikey.de/de/products/detail/traco-power/TSRN-1-2450SM/9383778
*IC1*	*Surge Protector SMD LTC4368*	1	5,87 €	5,87 €	https://www.mouser.de/c/?q=584-LTC4368IMS2PBF
*L100_ADC1, L100_ADC2, L101_ADC1, L101_ADC2, L102_ADC1, L102_ADC2, L103_ADC1, L103_ADC2*	*Inductor SMD 0805 10uH*	8	0,08 €	0,66 €	https://www.mouser.de/c/?q=810-MLZ2012N100LT000
*LWL100_ADC1, LWL100_ADC2, LWL103_ADC1, LWL103_ADC2*	*Fiber Optic Transmitter AFBR-1624Z*	4	18,04 €	72,16 €	https://de.rs-online.com/web/p/faseroptik-sender/8019207
*LWL101_ADC1, LWL101_ADC2, LWL102_ADC1, LWL102_ADC2*	*Fiber Optic Receiver AFBR-2624Z*	4	20,39 €	81,56 €	https://de.rs-online.com/web/p/faseroptik-empfanger/8019210
*P1_PWM_OUT1, P1_PWM_OUT2, P1_PWM_OUT3, P1_PWM_OUT4, P1_PWM_OUT5, P1_PWM_OUT6, P1_PWM_OUT7, P400*	*BNC-Connector*	8	4,71 €	37,68 €	https://de.farnell.com/search?st=1712355
*P2_PWM_OUT1, P2_PWM_OUT2, P2_PWM_OUT3, P2_PWM_OUT4, P2_PWM_OUT5, P2_PWM_OUT6, P2_PWM_OUT7, P401*	*SMA-Connector*	8	7,38 €	optional	https://www.mouser.de/c/?q=571-1-1478979-0
*P3, P405*	*3-Pin Jumper*	1	0,84 €	0,84 €	https://www.mouser.de/c/?q=798-MDF79P254DSA55
*P300*	*Header 15X2*	1	3,30 €	3,30 €	https://www.mouser.de/c/?q=798-MDF7P20DP254DSA4
*P301*	*PSK 2-Pin*	1	0,84 €	0,84 €	https://www.mouser.de/c/?q=798-MDF79P254DSA55
*P302*	*Header 10X2*	1	3,30 €	3,30 €	https://www.mouser.de/c/?q=798-MDF7P20DP254DSA4
*P303*	*Header 8X2*	1	3,30 €	3,30 €	https://www.mouser.de/c/?q=798-MDF7P20DP254DSA4
*P402, P403, P404*	*2-Pin Jumper*	3	0,84 €	2,52 €	https://www.mouser.de/c/?q=798-MDF79P254DSA55
*P_1*	*Banana plug 4 mm red*	1	3,08 €	3,08 €	https://www.mouser.de/c/?q=845-973582101
*P_GND1*	*Banana plug 4 mm black*	1	3,08 €	3,08 €	https://www.mouser.de/c/?q=845-973582100
*Q1, Q2*	*MOSFET SMD IRF8714*	2	0,63 €	1,26 €	https://www.mouser.de/c/?q=942-IRF8714TRPBF
*Q200, Q400*	*Bipolar Transistor SMD BC846*	2	0,13 €	0,25 €	https://www.mouser.de/c/?q=863-BC846BLT1G
*Q201*	*MOSFET SMD IRF7416*	1	0,86 €	0,86 €	https://www.mouser.de/c/?q=942-IRF7416TRPBF
*R2*	*Resistor SMD 0805 3 M9*	1	0,24 €	0,24 €	https://www.mouser.de/c/?q=71-RCV08053M90FKEA
*R3,R4*	*Resistor SMD 0805 150 K*	2	0,09 €	0,18 €	https://www.mouser.de/c/?q=603-RT0805FRE07150KL
*R5*	*Resistor SMD 0805 22 K*	1	0,09 €	0,09 €	https://www.mouser.de/c/?q=603-RT0805FRE0722KL
*R7*	*Resistor SMD 0805 1 K3*	1	0,09 €	0,09 €	https://www.mouser.de/c/?q=603-RT0805FRE071K3L
*R100_ADC1, R100_ADC2, R101_ADC1, R101_ADC2*	*Resistor SMD 0805 4 K7*	4	0,09 €	0,36 €	https://www.mouser.de/c/?q=603-RT0805FRE074K7L
*R200*	*Resistor SMD 0805 1 K*	1	0,09 €	0,09 €	https://www.mouser.de/c/?q=603-RT0805FRE071KL
*R201, R202, R301, R302, R303, R404*	*Resistor SMD 0805 10 K*	6	0,04 €	0,24 €	https://www.mouser.de/c/?q=603-RT0805FRE0710KL
*R203*	*Resistor SMD 0805 300 K*	1	0,09 €	0,09 €	https://www.mouser.de/c/?q=603-RT0805FRE07300KL
*R204, R304, R305*	*Resistor SMD 0805 120R*	3	0,09 €	0,27 €	https://www.mouser.de/c/?q=603-RT0805FRE07120RL
*R300*	*Resistor SMD 0805 240R*	1	0,09 €	0,09 €	https://www.mouser.de/c/?q=603-RT0805FRE07240RL
*R400*	*Resistor SMD 0805 2 K2*	1	0,09 €	0,09 €	https://www.mouser.de/c/?q=603-RT0805FRE072K2L
*R401, R1*	*Resistor SMD 0805 0R*	2	0,13 €	0,26 €	https://www.mouser.de/c/?q=71-CRCW08050000ZSTC
*R402, R403*	*Resistor SMD 0805 510R*	2	0,09 €	0,18 €	https://www.mouser.de/c/?q= 603-RT0805FRE07510RL
*R405*	*Resistor SMD 0805 300R*	1	0,09 €	0,09 €	https://www.mouser.de/c/?q=603-RT0805FRE07300RL
*S1*	*154Series-FuseHolder*	1	2,24 €	2,24 €	https://www.mouser.de/c/?q=576-01550900M
*U300*	*NOR Flash S25FL256SAGMFIR01*	1	6,33 €	6,33 €	https://de.farnell.com/search?st=2363330
*U301*	*EEPROMs with EUI-48 24aa02e48t-i/ot*	1	0,26 €	0,26 €	https://www.mouser.de/c/?q=579-24AA02E48TIOT
*U400*	*Optocoupler ACPL-K64L-560E*	1	5,81 €	5,81 €	https://www.mouser.de/c/?q=630-ACPL-K64L-560E
	*Arty Z7-20*	1	328,48 €	328,48 €	https://www.digikey.de/en/products/detail/410-346-20/1286-1152-ND/6674366
				**619,22 €**	

**Designator**	**Component**	**Number**	**Cost per unit**	**Total cost**	**Source of materials**
*ADC PCB*	*Printed Circuit Board*	1	35,64 €	35,64 €	https://www.multi-circuit-boards.eu
*AD1*	*ADC LTC1864*	1	16,74 €	16,74 €	https://www.digikey.de/de/products/detail/analog-devices-inc/LTC1864ACMS8-PBF/890875?s=N4IgTCBcDaIDIBUDCBGAHANgCwEEkFkBlNAYgAUAhAMQFoA5AERAF0BfIA
BOX1 *Cover*	*Top Cover 26.8 mm x 26.8 mm x 3 mm (L/W/H)*	1	3,16 €	3,16 €	https://www.mouser.de/ProductDetail/Wurth-Elektronik/36003250?qs=c50eh9DPO4PkJIJargT%2F7A%3D%3D
BOX1	*THT Frame 26 mm x 26 mm x 3 mm (L/W/H)*	1	4,58 €	4,58 €	https://www.mouser.de/ProductDetail/Wurth-Elektronik/36503255?qs=c50eh9DPO4NljY%2FcN0zQfQ%3D%3D
*C1, C5, C7, C9, C11, C202, C204*	*Capacitor SMD 1206 10uF 25 V X7R*	7	0,55 €	3,84 €	https://www.mouser.de/ProductDetail/TDK/C3216X7R1E106K160AB?qs=NRhsANhppD%2FkXRaItm%2FiQA%3D%3D
*C4, C6, C8, C10, C18, C19, C20, C21, C22, C23, C24, C25*	*Capacitor SMD 0805 1uF 50 V X7R*	12	0,29 €	3,46 €	https://www.mouser.de/ProductDetail/KEMET/C0805C105K5RACTU?qs=iP0bYSAMAFrBrUflcErrLQ%3D%3D
*C16, C17*	*Capacitor SMD 0805 150pF 150 V C0G*	2	0,26 €	0,52 €	https://www.mouser.de/ProductDetail/KEMET/C0805C151J1GACTU?qs=vSqmnp4Py0kAgppFfx08Gg%3D%3D
*C200, C203, C205, C206*	*Electrolyte-Capacitor SMD 470uF 35 V*	4	1,38 €	5,52 €	https://www.digikey.de/de/products/detail/panasonic-electronic-components/EEE-FK1V471AQ/1879860?s=N4IgTCBcDaIAoGECiAWAHANjAgKgWgDkAREAXQF8g
*D1, D3, D4, D5*	*LED SMD green*	4	0,62 €	2,49 €	https://www.mouser.de/ProductDetail/Kingbright/AP2012MGC?qs=m7sAZ2agqK%2FcFngWa9%2F3hQ%3D%3D
*D2*	*Diode SMD 85 V BAV199*	1	0,21 €	0,21 €	https://www.mouser.de/ProductDetail/Diodes-Incorporated/BAV199-7-F?qs=AV7Tsb4h%2F8A7dv79VWzRHQ%3D%3D
*D200, D201*	*Gas Discharge Tube 6.5 kV CG3 6.5*	2	4,01 €	8,02 €	https://www.mouser.de/ProductDetail/Littelfuse/CG36.5LD004?qs=f4GTrGqs%2FXNty75BewK6CA%3D%3D
*DCDC1*	*DC/DC Converter TSRN 1-2450SM*	1	10,54 €	10,54 €	https://www.digikey.de/de/products/detail/traco-power/TSRN-1-2450SM/9383778?s=N4IgTCBcDaIAQIQFQMoCUBycCMBaMALAKwAMKAsiALoC%2BQA
*L1, L2, L3, L4, L5, L7, L8*	*Inductor SMD 0805 10uH 500 mA*	7	0,09 €	0,65 €	https://www.mouser.de/ProductDetail/TDK/MLZ2012N100LT000?qs=%2FPzWLGNeQ%252BhBF9df2MjAZQ%3D%3D
*L200, L202*	*Inductor SMD 1210 330uH 1.4A*	2	0,69 €	1,38 €	https://www.mouser.de/ProductDetail/Bourns/SRR1210-331M?qs=4vvWAaIu%2Fq7%2FQuBPKlcNRQ%3D%3D
*OPT1, OPT4*	*Fiber Optic Transmitter AFBR-1624Z*	2	18,04 €	36,08 €	https://de.rs-online.com/web/p/faseroptik-sender/8019207
*OPT2, OPT3*	*Fiber Optic Transmitter AFBR-2624Z*	2	20,39 €	40,78 €	https://de.rs-online.com/web/p/faseroptik-empfanger/8019210
*P1*	*Coaxial flange socket*	1	5,49 €	5,49 €	https://de.farnell.com/multicomp/19-70-1-tgg/stecker-sma-ende-sternt/dp/1342652
*P200*	*Banana plug 4 mm red*	1	3,12 €	3,12 €	https://www.mouser.de/c/?q=845-973582101
*P201*	*Banana plug 4 mm black*	1	3,18 €	3,18 €	https://www.mouser.de/c/?q=845-973582100
*P202, P203*	*PSK 3-Pin*	2	0,23 €	0,46 €	https://www.mouser.de/ProductDetail/Molex/22-23-2031?qs=ILqg114nvd41XyIAFDpXfw%3D%3D
*R1, R2, R5*	*Resistor SMD 0805 10 K*	3	0,04 €	0,12 €	https://www.mouser.de/c/?q=603-RT0805FRE0710KL
*R3*	*Resistor SMD 0805 20 K*	1	0,09 €	0,09 €	https://www.mouser.de/ProductDetail/YAGEO/RT0805FRE0720KL?qs=Fz%2FrpjPuTcFiPdbHqrClXQ%3D%3D
*R4*	*Resistor SMD 0805 47 K*	1	0,09 €	0,09 €	https://www.mouser.de/ProductDetail/YAGEO/RT0805FRE0747KL?qs=Fz%2FrpjPuTcHR%2FQZkx3r7Fw%3D%3D
*R6*	*Resistor SMD 0805 0R*	2	0,13 €	0,26 €	https://www.mouser.de/c/?q=71-CRCW08050000ZSTC
*R7, R8*	*Resistor SMD 0805 4 K7*	4	0,09 €	0,36 €	https://www.mouser.de/c/?q=603-RT0805FRE074K7L
*R9, R10*	*Resistor THT 1G 10 kV*	2	4,47 €	8,94 €	https://www.mouser.de/ProductDetail/Ohmite/SM104031007FE?qs=xerK1%2Fx51jiqALsU1oTIVw%3D%3D
*R11*	*Resistor SMD 0805 510R*	2	0,09 €	0,18 €	https://www.mouser.de/c/?q= 603-RT0805FRE07510RL
*R12, R13*	*Resistor SMD 0805 1 K3*	1	0,09 €	0,09 €	https://www.mouser.de/c/?q=603-RT0805FRE071K3L
*R14*	*Resistor SMD 0805 390R*	1	0,09 €	0,09 €	https://www.mouser.de/ProductDetail/YAGEO/RT0805FRE07390RL?qs=%2FgLOArm3Wr4KJrVuTb07CQ%3D%3D
*U2*	*JFET Opamp SMD TL072*	1	0,81 €	0,81 €	https://www.mouser.de/ProductDetail/Texas-Instruments/TL072BCDR?qs=odmYgEirbwyOLHKvwmd6sw%3D%3D
*U3*	*Voltage Reference SMD 5 V ADR445*	1	10,66 €	10,66 €	https://www.mouser.de/ProductDetail/Analog-Devices/ADR445BRZ?qs=WIvQP4zGanj9lriDHL0gUg%3D%3D
*U200*	*Isolated DC/DC Converter REC6-1212SRW/R10/A*	1	48,84 €	48,84 €	https://www.mouser.de/ProductDetail/RECOM-Power/REC6-1212SRW-R10-A?qs=YWgezujkI1IKdqPlDSESPQ%3D%3D
*U201*	*Isolated DC/DC Converter REC6-1215DRW/R10/A/X1*	1	59,87 €	59,87 €	https://www.mouser.de/ProductDetail/RECOM-Power/REC6-1215DRW-R10-A-X1?qs=Lu0AfTIgUNP%2FbxCiNYxEQg%3D%3D
				**316,26 €**	

## 5 Build instructions

### 5.1 Safety measures

The hardware presented in this document must only be assembled and operated by trained personnel. The assembly includes the use of soldering irons, which can cause burns. The solder may contain harmful substances and the resulting solder fumes are harmful and should not be inhaled. Personal protective equipment should be used if necessary. The presented electronics themselves do not generate voltages above 24 V when properly assembled. However, when using IMS, high voltages of several thousand volts are used for the drift voltage which are applied to parts of the ADC boards. High voltages should only be handled by trained personnel. All legal requirements and occupational safety regulations must be observed at all times.

### 5.2 Printed circuit board assembly

To ensure proper installation of Surface-Mounted Devices (SMD) on the printed circuit boards (PCBs) of the mainboard and the ADC-board, it is recommended to use a stencil and solder paste. If this is not possible, solder small SMD components first and then solder larger through-hole components. The trigger pulse outputs/ inputs and the trigger pulse output/ input of the IMS mainboard can be equipped with either a Bayonet Neill Concelman (BNC) socket or a Sub-Miniature Version A (SMA) socket. Please refer to the descriptions below for the different configurations. It is important to note that not all parts of the electronics are mandatory.

### 5.3 Protection circuit

The power supply input of the hardware is equipped with a fuse, overvoltage/ undervoltage protection and reverse polarity protection. The range for overvoltage and undervoltage protection is set between 7.5 V and 14 V by selecting the resistor values of R1 = 0 Ω, R2 = 3.9 MΩ, R3 = 150 kΩ, and R4 = 150 kΩ according to the schematics. The jumper position P3 can be set to bypass only the protection Integrated Circuit (IC) or all protection. However, it is recommended to install and use at least the fuse.

### 5.4 Trigger input

The trigger input for coupling with external laboratory hardware can be configured in three ways. When one of the trigger input circuits is selected with jumper P405, the input signal is isolated by an optical isolator ACPL-K64L-460E from Broadcom to achieve galvanic isolation between the external laboratory hardware and data acquisition*,* and to protect the expensive SoC from overvoltage. In this case, jumpers P402 and P403 must be removed. The isolation voltage has been tested up to 500 V DC 10 s (SI 1) but the hardware is not meant to be used with high-voltage trigger inputs. By selecting “Trigger 1” the desired input voltage can be set by using the corresponding resistor values from Table 1. The resistor R401 must be removed for using this trigger input. In addition, selecting “Trigger 2” enables the use of an electric input circuit that supports an input voltage range of 4 V to 34 V, but introduces an input voltage-dependent trigger delay. To select this trigger mode, remove R402 and R403 and short-circuit R401. It is also possible to connect the input pin directly to the FPGA by short-circuiting jumpers P402 and P403 and removing jumper P405. In this case, the FPGA inputs are not protected against overvoltage. Therefore, the input signal must not exceed 3.3 V.

**Table 1:** Input resistor values (SMD 0805 resistors).

Input Voltage	R402	R403
3.3 V	430 Ω	430 Ω
5 V	845 Ω	845 Ω
12 V	2300 Ω	2300 Ω
24 V	4900 Ω	4900 Ω

### 5.5 Fiber-optic transceivers and receivers

**Fig. 3:**
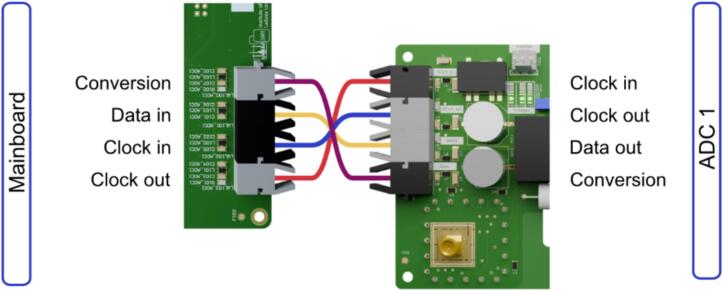
Overview of the optical transceivers to be connected between the mainboard and the ADC-board.

At least two transmitters and two receivers of the first ADC must be installed. The second ADC's transmitters and receivers can be retrofitted as needed. The fiber-optic cables have to be connected according to Fig. 3.

After the assembly, connect at least one ADC and a 12 V power supply with a minimum of 1 A to the mainboard. Do not connect the Arty Z7 board yet. In this case, the mainboard and the ADC should draw about 100 mA (5 W).

### 5.6 Preparation Arty Z7

**Fig. 4:**
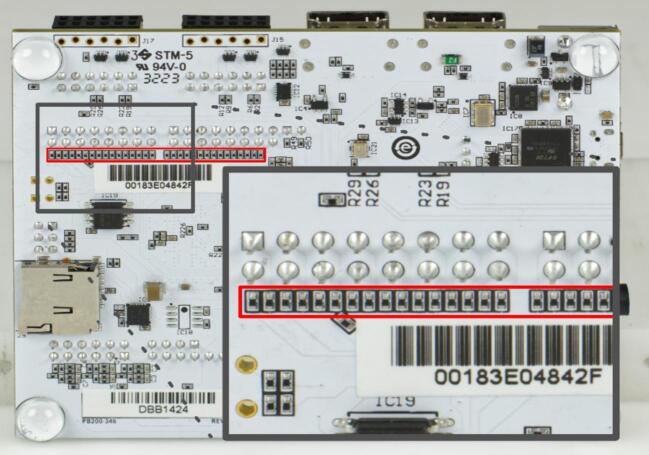
Backside of the ArtyZ7 board. The marked resistors have to be removed and short-circuited.

To prepare the Arty Z7 board, the marked resistors in Fig. 4 have to be removed and short-circuited. This can be done with a fine soldering iron and fluxless solder.

**Fig. 5:**
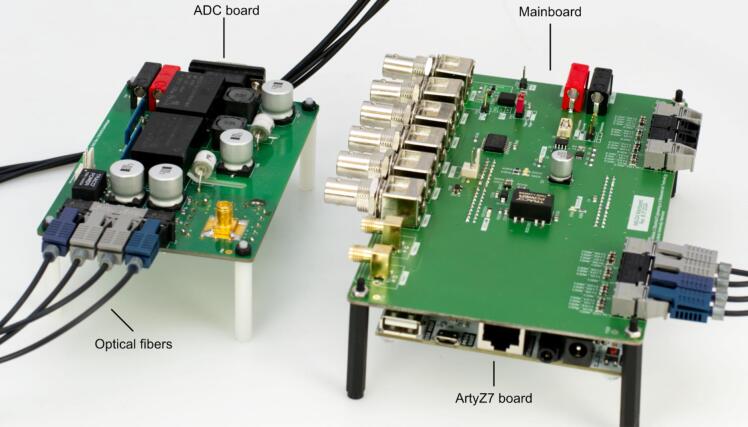
Fully assembled acquisition hardware with the Arty Z7 board plugged in on the bottom side of the mainboard.

To program the Xilinx Zynq xc7z020, set jumper JP5 to USB and JP4 to JTAG on the ArtyZ7 board. Connect the board with the “Prog UART” USB port to a computer. Open Xilinx Vivado 2023.2 and select “Flow → Open Hardware Manager”. Click on “Open Hardware” and select “Auto Connect”. A Digilent instance with an xc7z020_1 should be found. Right-click on xc7z020_1 and select the appropriate Quad-SPI memory device under “Add Configuration Memory Device”. For Rev. D of the Digilent ArtyZ7 board, search for “w25q128″ and select ”w25q128fv-qspi-x4-single“. Select the provided configuration file ”boot.bin“ and select the first stage boot loader file ”fsbl.elf“. The bin offset must be 0. Select ”Configuration File Only“ under Address Range and select ”Erase“, ”Program“ and ”Verify“. Click okay and wait for ”Flash programming completed successfully“. Disconnect the development board from USB and set JP4 to ”QSPI“ and JP5 to ”Reg“. Attach the ArtyZ7 board to the bottom of the mainboard using the header pins as shown in Fig. 5 and plug in the Ethernet cable connected to the network.

### 5.7 Debug interface

**Fig. 6:**
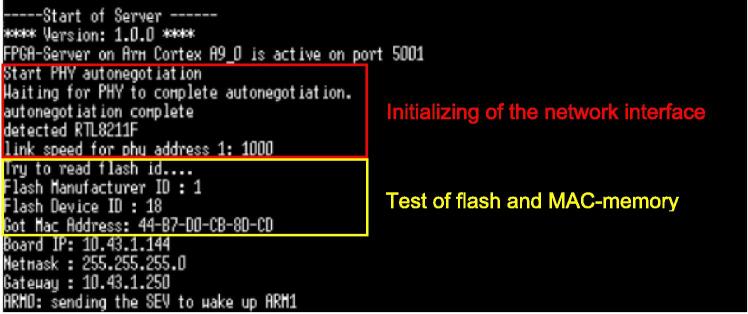
Boot sequence captured from the serial debug interface.

To check whether the firmware has been installed correctly and the network connection has been recognized, the debug console can be checked after starting the hardware using the virtual serial interface with the settings (Baud rate 115200, 8-bit, no parity, 1 stop bit, no flow control). After connecting to the system via the serial monitor and restarting the system with the “SRST” button on the Arty-Z7 board, the initial boot sequence should appear on the debug interface. During this boot sequence, the presence of the Flash Memory and the MAC Memory is checked. If everything is found successfully, the boot sequence looks similar to Fig. 6.

## 6 Operation instructions

### 6.1 IMS control

The TCP/IP communication protocol of the data acquisition is included in the design files and the use of this flexible communication protocol allows easy integration of the acquisition hardware into existing lab infrastructure. A reference implementation of this communication protocol is implemented in C++ and published under [34]. This software provides a complete measurement platform, including data storage in the open source container file format HDF5 [35] and a corresponding file viewer. Additionally, HDF5 is compatible with many other widely used software programs, such as Python and MathWorks MATLAB. The following operating instruction uses the software to demonstrate the functionality of the presented platform.

**Fig. 7:**
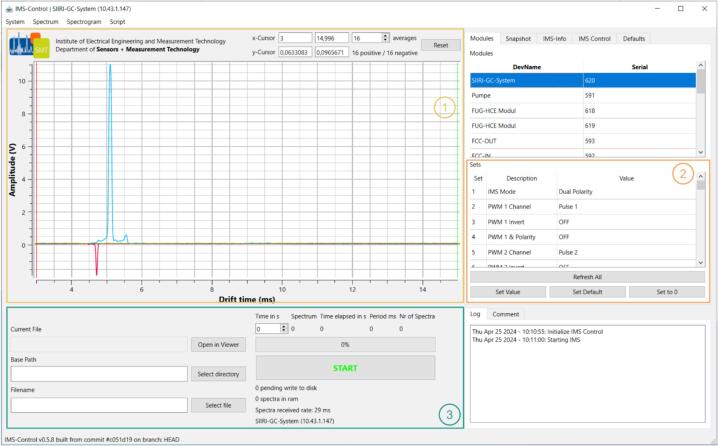
Control software for the IMS data acquisition. Reference implementation of the protocol in C++ including spectrum storage functionality and corresponding file viewer. Section 1: Received spectrum from the data acquisition. Section 2: Manipulation of the parameters of the data acquisition. Section 3: Selection of the memory file and start of continuous data recording.

When the control software is started, the entire subnetwork is scanned and all systems within this subnetwork are listed. To scan a different subnetwork, set the subnet prefix under “prefix”. If the system is directly connected to the computer, select the specific IP address (192.168.1.42), in all other cases, the acquisition hardware will receive an IP address through DHCP. After a successful connection, the recorded live spectra should be visible in the software as shown in Fig. 7. If no spectrum is visible, please check the fiber-optic cable connections and ensure that all transceivers and receivers are functioning properly. The FPGA firmware is supplied with default values for each setting, ensuring initial operation. Each setting of the data acquisition is divided into a parameter, which can be manipulated in the “Modules” tab (Fig. 7 section 2).

### 6.2 IMS modes

The system can be operated in different “IMS Modes” which can be selected with parameter 1. The “Positive Polarity” and “Negative Polarity” modes use ADC 1 to digitize the spectra. When selecting the polarity, the spectrum header, which is sent with every transmitted spectrum, is updated accordingly. In “Auto Polarity Switch” mode, the positive spectrum is always recorded before the negative spectrum. However, both spectra are sent together as one package to the measurement software. The alternating polarity signal inside the FPGA can be routed to one of the pulse outputs and can be used, for example, for ultra-fast polarity switching of the IMS [28]. In “Dual Polarity Mode”, both ADCs record simultaneously, and the spectra are sent as one package. This is useful, for example, for dual polarity IMS [22]. The modes “Positive ADC 1” and “Negative ADC 2” record either only the positive spectrum with ADC 1 or the negative spectrum with ADC 2.

Each spectrum recorded by the FPGA is assigned an incrementing spectrum number. The data is then stored in a ring buffer. The header of each spectrum sent to the computer includes the spectrum number of the transmitted spectrum, and the most recent spectrum number from the ring buffer to determine the number of spectra waiting in the RAM of the acquisition hardware.

### 6.3 Acquisition parameters

When operating a drift time IMS, the first operating parameter to be selected is the period time of the shutter. This value can be set with parameter 23 called “Period Time”. In the case of an IMS with a beam-chopping shutter, the cycle time can be chosen based on the slowest expected mobility *K* plus a margin of error. The maximum drift time *t_drift_* can be calculated using formula (1) based on the drift length *L* and the drift voltage *U_D_*.

(1) $$t_{drift}=\frac{L^{2}}{K∙U_{D}}$$

In the case of a field-switching shutter, the cycle time must also be selected based on the reaction rate of the formation of reactant and analyte ions [36]. This time has a major effect on the detection limits of a field-switching IMS.

Using formula (1) with the highest expected mobility *K*, the shortest expected drift time can be also calculated. Since no information can appear in the spectrum before this calculated drift time, a sample delay can be set in parameter 119. The data acquisition by the ADCs will start after this delay in each acquired spectrum.

To estimate the sampling rate of the ADCs to adequately represent a spectrum, two criteria must be met. First, it is necessary to ensure that the Nyquist-Shannon criterion is met [37] to avoid aliasing effects. For this reason, the sampling rate must be at least twice the stop frequency of the amplifier. For an IMS with an amplifier with a bandwidth of, for example, 30 kHz, a sampling frequency of 60 ksps would be sufficient. In addition, there should be sufficient data points on a Gaussian peak to be able to determine the correct peak maximum and peak width. If a Gaussian peak should have at least *N* sample points (in general 7–10) distributed over six times the standard deviation of the peak (99,7 %), the minimum sampling interval *t*_sample,min_ can be determined using the formula (2), based on the drift voltage *U*_D_*,* drift length *L*_D_, the highest mobility *K*, and the expected maximum resolving power *R*_p_.

(2) $$t_{sample,min}=\frac{6}{N-1}∙\frac{L_{D}^{2}}{2∙\sqrt{2∙ln(2)}{∙R}_{P}∙K∙U_{D}}$$

The sample interval and thus the sampling rate of the ADCs can be set with parameter 118. The sampling interval can be specified within a range of 4 µs to 1 ms, with the maximum sampling rate of 250 ksps corresponding to the shortest sampling interval of 4 µs.

The parameter 120 allows the user to select the number of sample points per spectrum. All parameters can be changed independently of each other and are subjected to a plausibility check and, if necessary, adjusted by the acquisition hardware. In addition, parameter 116 can be used to activate a digital low-pass filter, which is applied to the captured spectrum. The 3 dB bandwidth of the digital low-pass filter can be set here. A value of 0 Hz disables the digital low-pass filter. Parameter 117 can be used to pre-average the acquired spectra within the acquisition hardware prior to transmission to the computer.

### 6.4 Trigger modes

The hardware provides five different trigger modes, which can be selected via Parameter 24 “Period Type”. The default setting is called “Internal”. This generates a periodical pulse sequence inside the FPGA, which is fed to all units within the FPGA and triggers the data acquisition and pulse generation. In this mode, the period time is set via parameter 23 “Period Time”. In mode “External rising” or “External falling”, a falling or rising edge at the trigger input is used as the internal period clock. In this mode, the period time is ignored and a pulse before the end of the recorded spectrum can retrigger all units in the FPGA. For this reason, the desired sample steps of the ADC must be selected according to the expected clock rate of the trigger input. In contrast, the “Blocked rising” and “Block falling” mode enforces the select “Period Time”, a retriggering of the IMS occurs only if a rising or falling edge is detected after the “Period Time” is elapsed. In the trigger mode “Blocked-High”, the IMS pulse is retriggered every “Period Time” if the trigger input remains at a high level.

The modes “Trigger rising” and “Trigger falling” are designed, for example, for use with a gas chromatograph. In these modes, the internal period clock is stopped until a falling or rising trigger is detected at the trigger input. After this initial trigger event, the FPGA will continue running with the internal period cycle. To reactivate the trigger, the parameter must be changed back to internal and then to the desired trigger mode.

### 6.5 ADC calibration

The firmware includes a pre-calibration of the ADC inputs based on the component values provided in the schematics. If a precise calibration is required or if the component values are changed, connect the ADC input to a variable voltage supply and a precise multimeter. Prior calibration, ensure that the slope is set to 1 and the offset to 0 for parameter 121 (ADC1) and parameter 122 (ADC2). To make these changes, right-click on the desired “Set” and select “Edit Set” in the IMS control. Calibration coefficients can be obtained by sweeping the input voltage and documenting the raw ADC values displayed in parameter 121 and parameter 122 and the measured voltage by the multimeter. The displayed input signal in the software is calculated from the raw ADC data, offset and slope using equation (3).

(3) $$Input=(Data_{ADC}∙Slope)+Offset$$

Using this equation, new slope and offset values can be calculated for each ADC calibration. Enter these new values in the “Edit Set” dialog of the corresponding parameter. Subsequently, it is necessary to restart the hardware for the changes to take effect.

### 6.6 Pulse generation

**Fig. 8:**
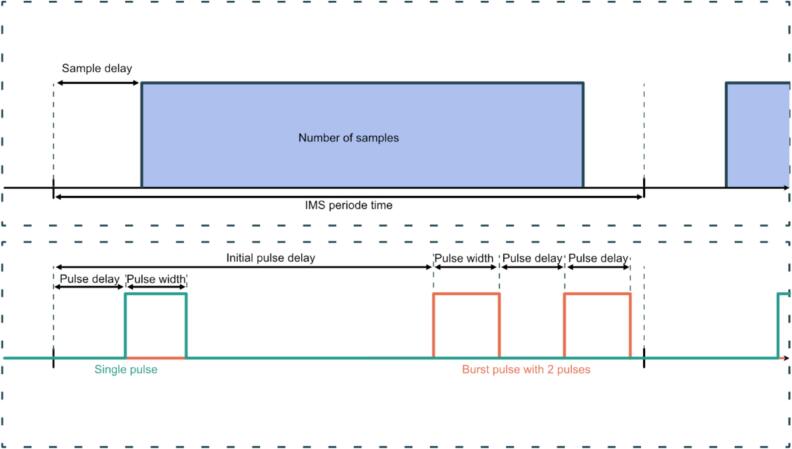
Exemplary illustration of the ADC timings and the pulse generation during one IMS period. As an example, for the pulse generation: a single pulse and a burst pulse with the burst size N = 2.

The presented hardware can output up to seven synchronous but independent trigger pulses via the BNC or SMA outputs. With the parameter “PMW X Channel”, the desired internal trigger pulse can be selected and is then multiplexed to the corresponding output on the PCB. Each output channel can be inverted and can be combined with the polarity signal through a logic “and” operation. Fig. 8 shows an example of these outputs. A total of 17 different trigger pulses are available for selection, seven single pulse generators, seven burst pulse generators, two virtual pulses and the IMS cycle pulse. The single pulse generator starts with each IMS period and can be delayed with the parameter “Pulse Delay”. The pulse “on time” can be specified with the parameter “Pulse Width”. For more complex pulse patterns, the seven available burst pulse generators can be used. With these burst pulse generators, the number of consecutive pulses can be selected with the “Burst Size” setting, while the initial delay of the sequence can be adjusted with the “Burst Init Delay” option. Each pulse of the burst pulse sequence is then defined by the “Burst Width” and “Pulse Delay” settings, which respectively determine the “on time” and “off time”. The “Burst Adj Width” setting increments each consecutive pulse width in the sequence by the specified value, while the “Burst Adj delay” increases the delay between two pulses within the sequence.

Additionally, it is also possible to combine pulses with different logical functions into one of the two available virtual pulses. In this mode, the logic operation can be selected with the “V Pulse Mode” option, and each pulse can be selected with the “On” or “Off” toggle. This mode enables the creation of more complex pulse patterns such as those used in FAT IMS [27].

## 7 Validation and characterization

### 7.1 Analog digital converter

**Fig. 9:**
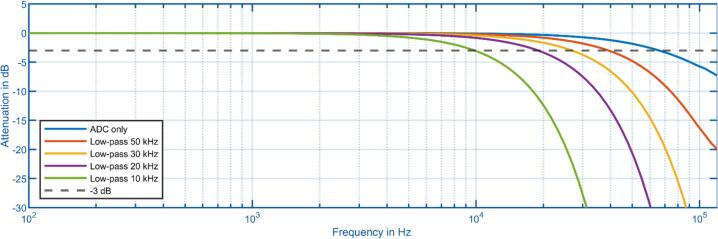
Measured frequency response of the isolated ADC reference implementation. The blue line represents the transfer function of the ADC with input filtering, while the other measurements show the results of combining the digital low-pass filter and the input filtering of the ADC board. (For interpretation of the references to colour in this figure legend, the reader is referred to the web version of this article.)

To validate the transfer function of the entire system, one ADC board was connected to the data acquisition. A Diligent DG 4202 waveform generator was connected to the input of the ADC board. The frequency response of the entire system shown in Fig. 9 was obtained by sweeping the frequency of a 20 V_PP_ sine wave and analyzing the transmitted waveforms. The blue line represents the raw ADC behavior without averaging or low-pass filtering. The other lines represent a low-pass setting on the acquisition hardware. The ADC itself with the analog low-pass circuit on the ADC board has a 3 dB bandwidth of 60 kHz. It can be concluded that in the standard configuration of the low-pass filter on the ADC board, a sampling frequency of at least 120 ksps should be selected, or alternatively, the signal should already be sufficiently band-limited by the amplifier to avoid aliasing. Since the measurements give the transfer function of the entire system, the transfer function of the digital filter and the analog input filter of the ADC are superimposed. Therefore, if a 3 dB cut-off frequency of 30 kHz is selected, the cut-off frequency of the overall system will occur at a lower frequency of around 27 kHz due to the effect of the input filter.

### 7.2 Pulse generation

**Fig. 10:**
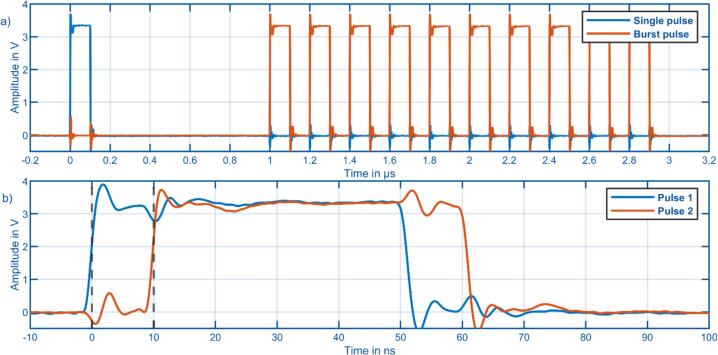
Validation of trigger pulse generation with a Rhode & Schwarz RTB2004 type oscilloscope. a) Single pulse and burst pulse sequence. b) Minimum temporal shift of 10 ns between two pulses.

To verify the trigger pulse generation, two of the pulse outputs of the data acquisition were connected to a Rhode & Schwarz RTB2004 oscilloscope. For pulse 1 a single pulse with a width of 100 µs and no delay was selected, while for pulse 2 a burst pulse with 10 pulses, 1 µs initial delay, 100 ns width, and 100 ns delay between each pulse was selected. As shown in Fig. 10 a), the measured timings correspond well to the values selected during data acquisition. Fig. 10 b) shows that the minimum possible shift between two pulses of 10 ns can be confirmed. The set pulse width of 50 ns is also reproduced correctly at the output.

### 7.3 Trigger input

**Fig. 11:**
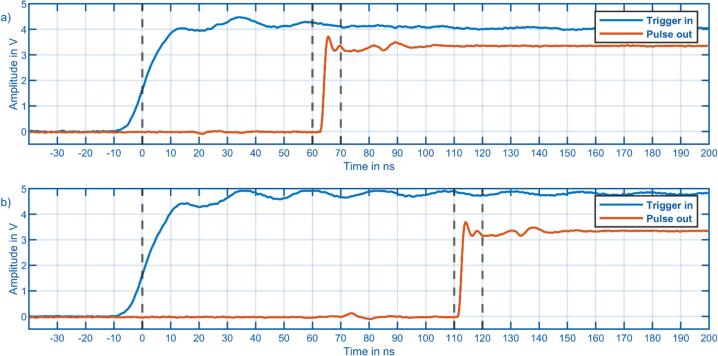
Measurement of trigger input latency with a Rhode & Schwarz RTB2004 type oscilloscope. a) Without optical isolation. b) Optical isolation with fixed resistor value.

When coupling the data acquisition with other laboratory equipment, such as lasers or gas chromatographs, it is important to minimize the trigger delay and ensure the delay is known. To measure the input delay, a trigger pulse was fed into the trigger input of the acquisition hardware. One pulse generator, without any delay, was routed out of one of the pulse outputs. This pulse will start when a rising edge is detected at the trigger input. Fig. 11 shows both signals recorded with a Rhode & Schwarz RTB2004, indicating a trigger delay of 60–70 ns without optical isolation.

When using the isolated input with fixed resistor values from Table 1, an additional 60 ns is added through the isolator IC, resulting in a total delay of 110–120 ns. For most applications, such as coupling with gas chromatographs, this delay is negligible, as GC runs generally take at least several minutes. However, if the IMS is triggered by a more timing critical device, for example, by a laser, it can be advantageous to know the exact trigger latency.

**Fig. 12:**
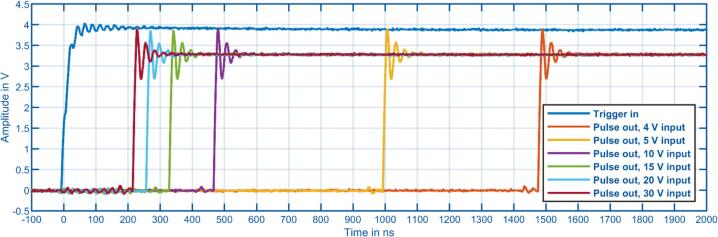
Measured input delay of the isolated variable voltage trigger input for various input voltage levels between 4 V and 30 V.

The delay of the isolated variable voltage trigger input was measured with different input voltages, as shown in Fig. 12. It can be observed that the delay decreases as the input voltage increases. This is caused by the input capacitance of the circuit, which is charged at different rates based on the input voltage. At the minimum input voltage of 4 V, the delay is 1.5 us, and it drops to around 200 ns at an input voltage of 30 V. This input is therefore suitable for interfacing with external laboratory instruments such as gas chromatographs, where a single-digit microsecond delay is negligible. However, this input provides a flexible input voltage range and does not need to be matched to the coupled external device.

### 7.4 Dual polarity spectrum

**Fig. 13:**
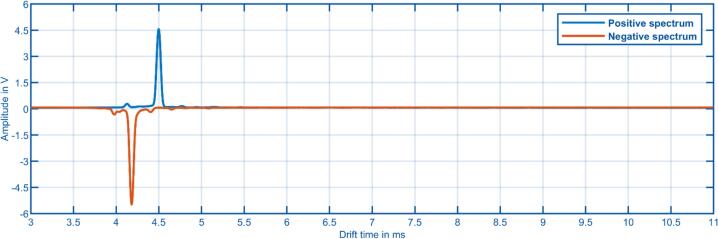
Positive and negative ion mobility spectrum of an axial dual polarity drift tube IMS with two ADCs in parallel for simultaneous recording. The IMS is operated with purified air just giving the positive and negative reactant ion peaks.

Finally, to demonstrate the functionality of the presented hardware, spectra of a dual polarity IMS and data of a GC-IMS are shown below. Fig. 13 shows the positive and negative ion mobility spectrum of an axial double drift tube IMS [22]. Here, the ion currents at the faraday detectors of both drift tubes were recorded simultaneously with the two ADCs. The IMS is operated with purified air and the ionization source is a soft X-ray source. The used extended field switching ion shutter [19] requires two synchronized pulse signals with different pulse lengths, which are provided by the acquisition hardware. The spectra were obtained by recording 2000 sample points per spectrum with a delay of 3 ms at a rate of 250 ksps and averaging the results over 1 s.

**Fig. 14:**
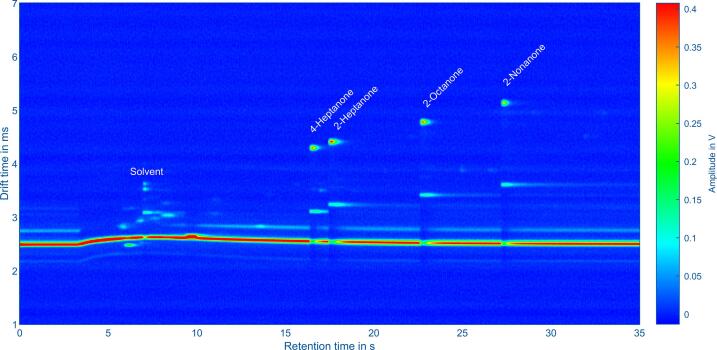
Topographic plot of GC-IMS data consisting of 3500 IMS spectra. Here, the trigger input of the acquisition hardware was used for synchronizing the GC and IMS. A mixture of 4-heptanone, 2-heptanone, 2-octanone and 2-nonanone in purified, dry air was analyzed.

In addition, Fig. 14 shows the topographic plot generated from the data of a GC-IMS. The GC is a HyperChrom GC with extremely narrow chromatographic peaks in the 100 ms range and short total analysis time below 60 s [38]. The IMS is a self-built device similar to [39] with a short drift length of just 41 mm and short drift times to sample the narrow chromatographic peaks. The IMS and data acquisition were triggered by the gas chromatograph using the trigger input of the acquisition hardware. A total of 3500 IMS spectra were acquired at 10 ms intervals and transferred to the control software. For demonstration, a compound mixture of 4-heptanone, 2-heptanone, 2-octanone, and 2-nonanone in purified, dry air was analyzed.

### CRediT authorship contribution statement

**Tim Kobelt:** Writing – original draft, Visualization, Validation, Software, Resources, Methodology, Investigation, Formal analysis, Data curation, Conceptualization. **Martin Lippmann:** Writing – review & editing, Resources, Methodology, Conceptualization. **Alexander Nitschke:** Writing – review & editing, Resources, Investigation. **Lou Kielhorn:** Software. **Stefan Zimmermann:** Writing – review & editing, Supervision, Resources, Funding acquisition, Conceptualization.

## Declaration of competing interest

The authors declare that they have no known competing financial interests or personal relationships that could have appeared to influence the work reported in this paper.
